# A tutorial in estimating the prevalence of disease in humans and animals in the absence of a gold standard diagnostic

**DOI:** 10.1186/1742-7622-9-9

**Published:** 2012-12-28

**Authors:** Fraser I Lewis, Paul R Torgerson

**Affiliations:** 1 Section of Epidemiology, VetSuisse Faculty, University of Zürich, Winterthurerstrasse 270, Zürich, Switzerland, CH 8057

## Abstract

Epidemiological methods for estimating disease prevalence in humans and other animals in the absence of a gold standard diagnostic test are well established. Despite this, reporting apparent prevalence is still standard practice in public health studies and disease control programmes, even though apparent prevalence may differ greatly from the true prevalence of disease. Methods for estimating true prevalence are summarized and reviewed. A computing appendix is also provided which contains a brief guide in how to easily implement some of the methods presented using freely available software.

## Introduction

Accurate estimation of the prevalence of disease is an essential part of both human and veterinary public health. For many pathogens this estimation is complicated by the lack of an appropriate reference test, that is, a diagnostic test which when applied to samples taken from a given target population has known accuracy (e.g. a gold standard/error free, or where the misclassification error is reliably known and understood). An important fact which is often overlooked is that the accuracy of a diagnostic test is a population specific parameter [1], as opposed to some intrinsic constant, as it depends upon the specific biological characteristics of the study population.

Despite the longstanding availability of approaches for estimating disease prevalence in the presence of diagnostic uncertainty, the use of such methods is still far from common. For example, a recent review of 69 prevalence studies [2] found that despite the lack of an available reference test, none of the studies provided either estimates of true prevalence or indications as to the accuracy of the diagnostics used.

When estimating disease status it is crucially important to distinguish between analytical and diagnostic accuracy of a test. Analytical accuracy is concerned with repeatability and robustness of the assay under laboratory conditions, when applied to samples usually with a known disease status [3]. In contrast, diagnostic accuracy is the ability of the assay to correctly identify a truly diseased subject from a non-diseased subject when applied to a sample from a randomly chosen individual from a given population of interest. This population may be defined in terms of biological characteristics, or else by geography or any other relevant commonality. High analytical accuracy does not imply high diagnostic accuracy. For example, a diagnostic test may reasonably be considered a gold standard test when applied to one study population but not another if these are epidemiologically different (e.g. different levels of disease exposure to additional pathogens or other biological confounders). Accuracy estimates provided by diagnostic test manufacturers should therefore be treated with considerable caution.

Statistical models which can accommodate both sampling error and misclassification error when analyzing data from imperfect diagnostic tests have been available in the literature for many years [1,4-7]. Key methodological articles include [8-13]. The development of more complex variants and extensions is still an active field of research [14,15]. More recently, the use of Bayesian and hierarchical statistical modelling has become increasingly common (e.g. [16-19]).

To date it is rare to find adjustment for diagnostic accuracy in regression analyses, for example in risk factor studies. This is, however, arguably just as important as in prevalence studies as when the diagnostic used is not perfect then the estimated effects of the covariates identified will have standard errors which are under-estimated (due to not incorporating the uncertainty resulting from diagnostic error). Moreover, such analyses identify those covariates related to apparent rather than true prevalence. Analogous methods to those reviewed here can also be used in regression analyses (e.g. see [20]).

In some studies the key parameter of interest may be disease prevalence, and estimates of the accuracy of the diagnostic tests used are simply nuisance parameters. Alternatively, it may be that the accuracy of the diagnostics themselves are of prime interest, for example if a new test has been developed and it is desired to examine whether it offers improved accuracy against existing tests, in which case disease prevalence is now a nuisance parameter. These two situations are mathematically identical, and the objective is to identify an appropriate statistical model which jointly estimates both disease prevalence and diagnostic test accuracy in the absence of a gold standard reference test.

We present here a brief overview of the key methods and concepts necessary to estimate disease prevalence and diagnostic test accuracy. While our focus is largely on veterinary public health, the accuracy of diagnostic tests used in veterinary medicine are highly variable, these methods apply equally to diseases of humans. Our objective is to provide an accessible, and non-technical introduction, to facilitate more widespread use of these techniques in analyses of data from epidemiological studies. We first present some basic definitions followed by thematic sections concerned with estimating disease status: i) within a randomly sampled individual; ii) within a single population group; and iii) across multiple population groups. We conclude with a brief discussion of the limitations and caveats required when using these techniques.

## Preliminaries

### Definitions

A number of technical terms and results are central to analyses involving imperfect diagnostic tests and we define some of these here. Sensitivity is defined as the probability that a diagnostic test is positive (*T*^+^) given that the sample being tested is known with certainty to be disease positive (*D*^+^). Similarly, specificity is the probability that a diagnostic test is negative (*T*^−^) given that the sample being tested is known with certainty to be disease negative (*D*^−^). We denote sensitivity and specificity as *S*_*e *_and *S*_*p *_respectively. Given these definitions:

(1) $$P(T^{+})=P(T^{+}|D^{+})P(D^{+})+P(T^{+}|D^{-})P(D^{-}),$$

(2) $$P(T^{+})=S_{e}\Pi +(1-S_{p})(1-\Pi )$$

which relates the probability of observing a positive test result, *P*(*T*^ + ^), to the true prevalence of disease in the study population, *Π*, and the sensitivity and specificity of the test. Two other important results are

(3) $$\text{PPV}:\;\;P(D^{+}|T^{+})=\frac{S_{e}\Pi }{S_{e}\Pi +(1-S_{p})(1-\Pi )},$$

(4) $$\text{NPV}:\;\;P(D^{-}|T^{-})=\frac{S_{p}(1-\Pi )}{\left(1-S_{e})\Pi +S_{p}(1-\Pi \right)}$$

where *P*(*D*^+^∣*T*^+^) denotes the positive predictive value, *PPV *, of the test, that is the probability that if the test is positive then the sample is truly disease positive. Similarly *P*(*D*^−^∣*T*^−^) is the negative predictive value, *NPV *. These results can be found in any standard epidemiology text (e.g. [21]).

The most commonly used probability model to describe sampling error when estimating disease prevalence is the binomial distribution. With *r* test positive subjects out of *n* tested, and *p* denoting the probability of observing a test positive subject then:

(5) $$P(\text{observe}\;r\;\text{test positive out of}\;n|p)=\left(\begin{array}{c} n\\ r \end{array}\;\right)p^{r}{\left(1-p\right)}^{n-r},$$

and combining this with equation (2) gives

(6) $$\begin{array}{l} P(\text{observe}\;r\;\text{test positive out of}\;n|p)=\\ \left(\begin{array}{c} n\\ r \end{array}\;\right)\;{\left(S_{e}\Pi \;+\;(1\;-\;S_{p})(1\;-\;\Pi )\right)}^{r}\;{\left(1\;-\;S_{e}\Pi \;-\;(1\;-\;S_{p})(1\;-\;\Pi )\right)}^{n-r}. \end{array}$$

The statistical model in equation (6) is arguably the simplest model for estimating disease prevalence and diagnostic error, although note that it is over-parameterized with three parameters (*Π*,*S*_*e*_,*S*_*p*_) but only one piece of information provided by the data (the apparent prevalence, *r*/*n*). As such, this model is only of practical use within a Bayesian context (see later) as this allows for additional (prior) information about *ΠS*_*e*_ and *S*_*p*_ to be used to supplement the observed data. Related to equation (6) is the classic Rogan-Gladen estimator of true prevalence in the presence of an imperfect diagnostic test [22]. This estimator has the advantage of simplicity but it requires that *S*_*e*_ and *S*_*p*_ are both known (and constant), which may be unrealistic in practice. In addition, the Rogan-Gladen estimator can produce estimates of prevalence which exceed one or are negative [23], (in which case the values of *S*_*e*_ and *S*_*p*_ must be incorrect for the current population of interest). Generally speaking, other more modern approaches such as those subsequently presented are preferable.

### Fitting models to data

Consider the simple model given in equation (6), and suppose we have observed data which comprises of *n *= 50 subjects tested, using some imperfect diagnostic, and of these *r *= 15 are test positive. We wish to use this information to estimate the true prevalence of disease in the population from which these *n *= 50 subjects were sampled. In general, fitting such a model to data typically requires writing (a few lines of) bespoke computer code in specialist software, e.g. WinBUGS/OpenBUGS [24] or JAGS [25], as opposed to relying on built-in functionality as would be the case with, for example, logistic regression modelling.

One of three broad numerical techniques is typically utilized. The classic approach is a direct application of maximum likelihood estimation [8]. An alternative is to use the Expectation Maximization (EM) algorithm [26], which is also a maximum likelihood approach but ideally suited to problems comprising latent class variables, which is exactly what we have here as the true disease status of each observation is only latently observed. Some details of how to implement EM estimation in the context of imperfect diagnostic tests are given in [27] and [20]. The third option is to use a Bayesian approach. There is a vast literature on Bayesian statistics, two widely used standard texts are [28] and [29]. A brief non-technical veterinary focused introduction to these three techniques can be found in [4].

A key distinction between Bayesian and maximum likelihood modelling is that in a Bayesian approach prior knowledge about likely values for the parameters in the statistical model must be included. There are two options here: i) non-informative priors, which in effect, do not incorporate any prior knowledge about the parameters of interest into the modelling process; and ii) “data-driven” priors in the sense that some evidence — external to the current study data — for example, expert opinion from appropriate specialists or existing relevant literature, is available about likely values for one or more of the parameters of interest. Note also that non-informative (e.g. vague) priors need not be on the range [0,1], for example, a Gaussian distribution with zero mean and large variance (e.g. 1000) may be appropriate when parameterizing prevalence or test accuracies in terms of covariates (e.g. the final example in the computing appendix). If a data-driven prior is chosen then it is absolutely essential that such a choice can be robustly justified, i.e. it is not some arbitrary guess, as this may have a strong influence on the modelling results obtained.

Considering the model in equation (6), in a Bayesian context we would need to additionally provide information/prior assumptions about likely values for the true prevalence in the population of interest, along with estimates of the test’s sensitivity and specificity. In practice the ability to include prior knowledge can be highly beneficial as it is common to be able to say at least something, even if this is very vague - provided it is justifiable - about the likely value of the true prevalence of a disease (e.g. < 80*%*) or the likely range of sensitivity and specificity for a given diagnostic. This prior information can be as strong or weak as required including, in effect, no prior knowledge where it is simply assumed that *Π*,*S*_*e*_ and *S*_*p*_ lie between zero and one and can be any value in between with equal probability. Generally speaking, the results from a Bayesian analysis which uses little or no prior knowledge often give results which are extremely similar to those from maximum likelihood.

Other than the obvious attraction of being able to incorporate prior information into any analyses, a Bayesian approach also has an important practical advantage: several high quality software packages (JAGS and OpenBUGS) are available which can be used with relative ease to fit Bayesian models appropriate for estimating true disease prevalence and imperfect diagnostic accuracy. Some bespoke computer code is still required, although this comprises mainly of defining the desired model in a way which can be understood by JAGS/OpenBUGS. While such models may be easy to fit to data, in common with all Bayesian modelling careful diagnostic and sensitivity analyses are essential [28].

The computing appendix contains a detailed guide in how to write code for fitting and comparing models for estimating disease prevalence and diagnostic accuracy in JAGS. Starting with the simple example given in equation (6), it is shown how this code can be readily extended to incorporate highly complex models. Also provided is how to choose between competing models using the Deviance Information Criterion (DIC) [30] - as model selection in any statistical analysis is crucial for ensuring robust results. While the DIC is very commonly used in Bayesian analyses, and is straightforward to estimate, it is not without its critics and its reliability in some situations is an active area of statistical research (e.g. [31]). The code required by OpenBUGS and JAGS is broadly similar, but a considerable attraction of JAGS is its very simple command line interface and that it is available for many different platforms (e.g. windows, mac and linux). For R [32] users the rjags library provides a way to run analyses in JAGS without leaving R, and works across all platforms, although this does still first require familiarity with the JAGS user manual.

## Statistical models for use with imperfect diagnostic tests

### Disease presence within an individual subject

If the diagnostic test used is not a gold standard, then on observing a positive test result for a given subject, the key question is how likely is it that this subject is truly disease positive? This is the *PPV* (equation 3) and its value may depend on many things, not least the true prevalence of disease within the population from which the particular subject has its provenance.

To show the considerable public health ramifications that failing to account for imperfect diagnostic accuracy can have, we present a very simple but real epidemiological example based on a recent legal case in the UK [33]. Consider a farm with 118 cattle undergoing routine surveillance for bovine tuberculosis (bTB) using the comparative intra-dermal skin test in the north east of England where bTB is rarely seen. One animal has a positive test result. The skin test has a sensitivity of ≈ 0.78 and specificity of ≈ 0.999 [34]. With an apparent prevalence of 1/118 the true herd prevalence *Π* using equation (1) can be estimated at ≈ 0.0096. There is available a secondary blood test for bTB based on an interferon gamma assay (IFNg). Because of the ongoing epidemic of bTB in the UK, DEFRA (Department for Environment, Food and Rural Affairs) has a policy of testing all animals with IFNg on a farm where bTB is confirmed providing the farm is in an area of the UK where bovine bTB does not usually occur [35]. Should one of the remaining 117 animals on the farm be positive to this secondary test a question that could be asked is what is the probability the animal has tuberculosis (i.e. the PPV of the secondary test)? The sensitivity and specificity of the interferon gamma test are reported as 0.909 and 0.965 respectively [36]. Therefore, in this case we have *Π *= 0.0096, *S*_*e *_= 0.909, *S*_*p *_= 0.965 and hence a PPV of 0.201. Therefore, the probability of a false positive is 0.799. This may be one reason why many cattle giving a positive INFg test result, originating from such low endemic districts, have no evidence of infection at post mortem [35]. This simple example demonstrates the dangers of incorrectly treating an imperfect diagnostic test as error free, or equivalently interpreting apparent prevalence as true prevalence.

Assessing the disease status of any individual subject is, to a greater or lesser extent, probabilistic in nature. As the above example highlights, however, when dealing with imperfect tests and diseases with low prevalence then the chance of observing a false positive result even with extremely specific diagnostic tests can be appreciable. It is therefore essential to always estimate the *PPV *.

### Disease prevalence within populations of subjects

One of the most well cited and founding articles in analyses of data from imperfect diagnostic tests is by Hui and Walter [8] who are credited with deriving rules for study designs which allow for the sensitivity and specificity of imperfect diagnostic tests, and the associated true prevalence of disease, to be estimated.

In short, using multiple imperfect diagnostic tests and one or more independent populations of animals, with differing prevalences, provides sufficient information to allow all model parameters to be estimated. Consider two examples: i) one population of 100 subjects are tested for disease, and each individual is tested using three different diagnostic tests (of uncertain accuracy), and it is assumed that the tests provide (biologically) independent results. This study design provides seven degrees of freedom (seven independent pieces of information), the counts of how many subjects out of 100 have each test pattern, e.g. suppose 15 individuals have $\left(T_{1}^{+},T_{2}^{+},T_{3}^{-}\right)$ — positive results for test 1 and test 2 but negative for test 3. There are eight possible patterns but since the total number of subjects is fixed then there are only seven independent counts. This study design has seven parameters which need to be estimated: *Π*, $S_{e_{1}}$, $S_{p_{1}}$, $S_{e_{2}}$, $S_{p_{2}}$, $S_{e_{3}}$, $S_{p_{3}}$ — the true prevalence plus the sensitivity and specificity of each test. Hence, we have seven parameters and seven degrees of freedom and therefore each parameter can be estimated as, generally speaking, it requires one degree of freedom to estimate each parameter in a model.

For a second example consider two independent populations (with assumed different prevalences), where each subject is tested using two imperfect (and assumed independent) tests. This time we have six parameters to estimate *Π*_1_, *Π*_2_, $S_{e_{1}}$, $S_{p_{1}}$, $S_{e_{2}}$, $S_{p_{2}}$, the prevalence in each population and the sensitivity and specificity of each test. In each population we have three degrees of freedom - three independent counts, one for each test pattern, $\left(T_{1}^{+},T_{2}^{-}\right)$ etc, so again can estimate all the parameters required.

In summary, by adding additional population groups and/or additional tests then the unknown diagnostic sensitivity and specificity, and true disease prevalence, can be estimated. A number of important caveats apply to these study designs. In particular, it may be unreasonable to assume that the diagnostic tests used will be independent as they may share a similar biological basis.

The Hui and Walter “rules” apply only to maximum likelihood estimation of the model parameters, technically speaking these criteria ensure the model is identifiable, that each parameter in the model can be uniquely estimated given only the observed data. See [37] for a detailed examination of identifiability in respect of models for imperfect diagnostic tests. It should also be noted that the Hui and Walter approach can perform very poorly in situations where the prevalences across different populations are similar [38]. When using a Bayesian approach the situation is more flexible as the use of prior information can allow all model parameters to be readily estimated [11]. For example, the model in equation (6) does not meet the Hui and Walter rules as it has only one test and one population, and with three parameters these cannot be estimated uniquely using maximum likelihood — but can be readily estimated in a Bayesian context provided sufficient prior information is available (subject to some technical caveats such as the use of a proper prior, see [28]).

#### Correlated Diagnostic Tests

In Hui and Walter is it assumed that the diagnostic tests being used were conditionally independent, e.g. given a known positive sample then

(7) $$\begin{array}{l} P(T_{1}^{+},T_{2}^{+}|D^{+})\;=\;P(T_{1}^{+}|D^{+})P(T_{2}^{+}|D^{+})\\ \;\;\;\;\;=\;S_{e_{1}}S_{e_{2}}. \end{array}$$

This is only tenable, however, if the tests are based on different biology, for example gross pathology and PCR, otherwise is it difficult to justify that each test provides independent evidence in support of the presence (or otherwise) of disease. Developing models which can incorporate dependence between test results comprises a large body of work, with one of the first examples being [9] followed by many others (e.g. [12,13,39,40]). The impact of assuming conditional independence between tests, or indeed assuming a particular dependency structure, is of crucial importance in such analyses [6,27,41] and we return to this later. There are a number of different ways to incorporate adjustment for correlation between tests, following [13] the basic idea is as follows:

(8) $$\begin{array}{ll} P(T_{1}^{+},T_{2}^{+}|D^{+})\;=\;P(T_{1}^{+}|D^{+})P(T_{2}^{+}|D^{+})\; & \;\\ \;\;\;\;\;=\;S_{e_{1}}S_{e_{2}}+\mathrm{co}v_{s},\; \end{array}$$

where compared to equation (7) an additional parameter is introduced whose purpose is simply to provide a numerical adjustment to ensure that the conditional probability $P\left(T_{1}^{+},T_{2}^{+}|D^{+}\right)$ is no longer equal to the product of the test sensitivities. The statistical price for introducing covariance terms (e.g. *cov*_*s*_) is that each one of these requires a degree of freedom in the study design and so additional populations and/or tests are required. How best to utilize the degrees of freedom available in any given study design is crucial in selecting an appropriate statistical model. Degrees of freedom can be “saved” by fixing or collapsing parameters in the model, for example by assuming that one or more of the tests are 100% specific or that two tests may have approximately the same specificity but different sensitivities.

We now present a brief empirical example comprising of multiple (three) imperfect and potentially correlated tests. All the JAGS (and R) code, and the data necessary to conduct this example, along with detailed instructions, can be found in the computing appendix (together with several other related examples). Consider the situation where we have one population of 200 subjects, and where each is tested once with three different diagnostic tests. We find that the (mean) apparent prevalence is 44% (88/200) and we wish to estimate the true prevalence. In terms of prior knowledge based on known biology and expert experience of the assays involved, we assume that the specificity for the third test is perfect (100%), and use prior Beta distributions for the specificity of the first and second tests of *Be*(9,1) for each, e.g. a mean of 90% accuracy and 2.5% and 97.5% quantiles of approximately 66.4% and 99.7% respectively. Non-informative, e.g. *Be*(1,1), priors are used for all other parameters. In other words, we are fairly confident that the specificity of the first and second tests will be reasonably good but we are not sure of exactly how good. We have no other evidence to assert prior knowledge into the modelling in respect of the other parameters. We also cannot discount (on biological grounds) covariance between the tests, and explicitly include a term in our model for covariance between the second and third tests when the subject truly has disease. Given the data, our prior assumptions (distributions) and our model structure, i.e. a multinomial model parameterised as three tests, one population and one covariance term, we can then use JAGS to produce an estimate of the true prevalence. We find using this particular model formulation that the mean true prevalence is 36.2% (see the computing appendix for detailed parameter estimates). What is also of some note is that if we were to assume that all these tests were conditionally independent (i.e. no covariance terms) then our mean estimate of the true prevalence drops to 18.3% (this example is also in the computing appendix). This highlights the crucial importance of model selection (as discussed later), and that it is essential to consider different covariance structures between tests, and then choose that which is most supported by the observed data.

### Disease prevalence across multiple population groups

In disease surveillance the objective is typically wider than estimating disease prevalence or diagnostic accuracy in respect of one or more independent population groups, but where estimates are desired across a large number of groups. This is particularly true when considering populations of food animals, where a main question of interest is the prevalence of disease in the national herd rather than on an individual farm. If multiple test results were available per subject/animal - which is uncommon due to the very considerable resources required - then such studies could be analyzed using the one population multiple test design (e.g. [27]). When considering populations structured into groups (i.e. farms or herds in the case of livestock), then issues such as within group correlation effects may need to be taken into account. In particular, what is typically desired is an estimate of the distribution of within-group (e.g. herd) disease prevalences based on observations from some random subset of individual groups.

In [19] a veterinary case study is presented utilizing a hierarchical model involving multiple herds and two conditionally independent tests, where the goal is to estimate the distribution of within-herd prevalences across many herds. A discussion of herd level testing in the absence of a gold standard diagnostic can also be found in [7]. A particularly important design of study is where only a single imperfect diagnostic test is used across many population groups, and such studies are amenable to analyzes which can be done in various ways. Using a hierarchical modelling approach such as the beta-binomial technique presented in [7] or alternatively using finite mixture modelling which seeks to identify distinct prevalence cohorts within a population [42]. While these are mathematically rather sophisticated models they are little more difficult to code in JAGS than other simpler models. Other ways to estimate the distribution of true prevalence across many population groups when only a single imperfect diagnostic test is available is to exploit laboratory replicates, as this can greatly increase the amount of data available in a study, but some care is required as replicates from the same subject will likely be correlated [43].

## Reliability and Validation

While methodological contributions and case studies estimating disease prevalence and the accuracy of diagnostic tests represents a sizable body of work, there are outstanding technical and conceptual issues. In particular, as the true prevalence is not directly observed but only latently observed (unlike apparent prevalence) such models are not testable against observed data without other additional information [27]. Many statistical models, however, comprise of latent parameters, which are a necessary part of their formulation. One very common example being linear mixed models [44], and methods for estimating such parameters are well established.

As with all statistical modelling, the resulting parameter estimates will only approximate nature’s true values (as the complete biological and physical mechanisms which generated the observed data will generally be unknown), and the estimates of these values will depend on the precise formulation of the chosen statistical model. It is, therefore, absolutely crucial to select the most appropriate model for given study data by comparing - possibly numerous - different competing models. This is particularly true when estimating such unobservable (latent) parameters as true disease prevalence and diagnostic accuracy. Assessing the impact of different assumptions in regard to dependence between tests or assumptions relating to the prevalence distribution across population groups, e.g. is a disease free cohort needed if many individual groups are free from disease, can be of considerable practical importance [19,27,42,43,45].

While prevalence estimation and diagnostic accuracy are strongly biologically driven it is still essential to perform robust model selection. The choice of sampling distribution is arguably less of an issue here than in other types of analyses, as generally speaking we typically have sets of dependent Bernoulli trials (although some studies do require additional measures such as zero inflation or over-dispersion for within group clustering). The model selection process here typically centres around choosing the optimal covariance structure between different diagnostic tests. For example, if there are three or four (or more) tests then there are a great many different covariance structures possible. Ideally we wish to determine that which is most optimal given the observed data. While biological knowledge can obviously be helpful here, this is, in practice, something of a challenge. A diagnostic test which is based on a serological assay may be reasonably considered (conditionally) independent from a diagnostic test which uses gross pathology, and so the relevant covariance parameters set to zero. This is, however, much more difficult to argue when different serological based tests are used together, or other tests which are based on similar biological mechanisms. It may be assumed apriori that such tests may be covariant (dependent), but that is rather different from whether the observed study data actually supports such an assertion. In order to determine an optimal (parsimonious) model then extensive model selection comparing the goodness of fit (e.g. DIC) across different covariance structures is essential. Not only because this is of interest in terms of the biological results, but also because this may have a substantive impact on the resulting estimates of the parameters of interest, e.g. prevalence and diagnostic accuracies.

There are very few simulation/validation studies in the literature, e.g. where the results of a model are compared with the “truth”. One example can be found in [27] who compare results from a model of a single sample of 666 observations and three imperfect tests, with results from a known gold standard test. The model used in this example under-estimates the true prevalence of disease (42% against 54%) and over-estimates the accuracy of the diagnostic tests. Another example, which uses different animals for the imperfect and gold standard comparison, can be found in [46].

A potential difficulty in assessing model robustness using simulation is that the parameters estimated may be all highly interdependent, and therefore how well any model performs may depend closely on the precise combination of (true) values used. This is particularly problematic when considering the general case of estimating prevalence across a group of populations, e.g. farms, as there are the additional parameters required to describe the shape of the within-herd prevalence distribution, and this may take almost any shape. Using simulation studies to draw general conclusions as to the likely situations in which some models may perform better than others is therefore a significant challenge, and may partly explain the lack of such studies in the literature.

Finally, before conducting any analyses it is essential to clearly define the disease status being examined, e.g. what constitutes a sample being disease positive, as without this, while the models can still be fitted and parameters estimated, the numerical results will have no meaningful biological interpretation.

## Conclusion

There is a broad and established literature on estimating the prevalence of disease in humans and animals in the absence of a gold standard diagnostic test. There is, therefore, little scientific justification for reporting apparent prevalence in place of true prevalence, and similarly assuming diagnostic tests are either gold standard tests or have known accuracy when this has not been established on the particular study population. The main practical obstacle in applying such techniques is that the analyses required are not pre-built into standard statistical software, however, using more specialist programs such as JAGS/OpenBUGS appropriate analyses can be conducted with relative ease.

## Competing interests

The authors have no financial or non-financial competing interests to declare.

## Authors’ contributions

FIL wrote the manuscript and developed the computing appendix, PRT co-wrote and assisted with the manuscript. Both authors read and approved the final manuscript.

## Funding

P. R. Torgerson received support from the Swiss National Science Fund CR3313_132482/1.
